# Waiting times in primary care depending on insurance scheme in Germany

**DOI:** 10.1186/s12913-018-3000-6

**Published:** 2018-03-20

**Authors:** Andres Luque Ramos, Falk Hoffmann, Ove Spreckelsen

**Affiliations:** Department of Health Services Research, Carl von Ossietzky University, Ammerländer Heerstrasse 140, 26111 Oldenburg, Germany

**Keywords:** Waiting times, Statutory health insurance, Private health insurance, Social inequality

## Abstract

**Background:**

Waiting times for an outpatient appointment in Germany differ between insurants of the statutory and private health insurance schemes, especially for specialised care. The aim of this study was to uncover possible differences in waiting times depending on health insurance scheme and to identify predictors for excessive waiting times in primary care.

**Methods:**

We used data of the Bertelsmann Foundation Healthcare Monitor, which is a repeated cross sectional study dealing with experiences in health care and attitudes towards current health policy themes. We analysed the surveys conducted from 2011 to 2013, with respondents assigned to their health insurance fund, namely AOK, BARMER GEK, BKK, DAK, TK, IKK, other statutory funds and private funds. The mean waiting times for an appointment and spent in a physician’s waiting room, and the satisfaction with waiting times were evaluated with respect to different health insurance funds. A logistic regression model was used to calculate the chance of excessive waiting times with respect to health insurance fund, age, sex, health and socioeconomic status. The ninetieth percentile of the waiting time distribution (10 days) was chosen as the cut-off point between average and excessive.

**Results:**

A total of 5618 respondents were analysed. Mean waiting times in primary care were low (4.0 days) and homogeneous (SHIs: 3.6–4.9 days), even though privately insured respondents reported shorter waiting times for appointments (3.3 days). They also reported a greater satisfaction with waiting times (77.5%) than SHI insurants (64.5%). However, we identified a group (10.1%), who experienced excessive waiting times in primary care. Compared to privately insured respondents, the chance of excessive waiting times was increased for SHI insurants (highest odds ratio for BKK: 2.17; 95%-CI: 1.38–3.42). Additionally, higher age and residence in East Germany were associated with higher chances of waiting times of 10 days or more.

**Conclusions:**

Primary care in Germany is readily accessible with generally short waiting times. However, barriers in access to the health care system affect a certain part of patients depending on insurance status, age and region of residence. Ways to improve the access need to be studied.

## Background

A major aim of all health care systems is the provision of equal access to health care for everyone [1]. Delayed access to health care seem to be related to poorer health in elderly and vulnerable people and leads to more preventable hospitalizations [2]. However, social inequalities in health and health care utilization can be found in nearly all European welfare states [3]. Such inequalities can also be observed in Germany [3–7]. A current review of social disparities in outpatient and inpatient care found that persons with lower socioeconomic status are disadvantaged in their access to health care (e.g. waiting times) [7]. Persons with higher socioeconomic status were more commonly treated by specialists and use preventive care more frequently. The authors also found differences in health care depending on the insurance type [7]. The German health insurance system is characterized by a dual structure of statutory health insurance (SHI) and private health insurance (around 10% of all people were insured privately) [8]. Only insurants with an income over a certain limit (2016: EUR 56,250 per year), the self-employed or public servants can switch from SHI to private insurance funds. The SHI comprises of several health insurance funds (118 in 2016). There are only minor differences between the benefits provided and the insurance fees. Traditionally (until 1996), every insurance fund had an occupation-specific population (e.g. engineers had a specific SHI). Since 1996 insurants can choose among nearly all SHI funds. However, there are still sociodemographic differences between the different insurance funds [9, 10]. In addition, the prevalences of diabetes, asthma, hypertension, neurodermatitis and musculoskeletal diseases varied between the funds [9, 10]. Several other population-based studies of children and adults also found differences in sociodemographic characteristics and health service use between the funds [11–13].

Two reviews from 2010 and 2016 stated that there are inequalities in health care depending on insurance status [7, 14]. Privately insured patients had a higher chance of being treated with innovative drugs [15]. The annual costs of antihypertensive pharmacotherapy were 35% higher for private health insurants than for SHI insurants [16]. Another study found differences in organ transplantation depending on insurance status [8]. Although only 10% of the German population is privately insured, privately insured patients received 15% of all transplanted organs and were classified with greater urgency than SHI insurants [8]. Privately insured respondents reported longer treatment durations and more attention to the needs and questions [17]. Mean waiting times were lower for privately insured persons than in SHI insurants, especially in specialised care [1, 18]. In a survey of 189 specialists in the Colone region mean waiting times for an allergy and pulmonary function test were 26.0 days for the privately insured and 36.7 days for SHI insurants [18].

A study by Roll et al. showed a moderate effect of income on waiting times in primary care (decreasing waiting times with increasing income). However, they did not study, whether waiting times also differ within different SHIs in primary care in Germany. We can assume that there are also differences in waiting times because of socioeconomic differences between the membership of, and different health burdens carried by, the various SHIs.

The aim of this study was to examine, whether there are insurance scheme-dependent differences in waiting times and satisfaction with waiting times in primary care, and to identify predictors for excessive waiting times.

## Methods

### Database

This study is based on data from the German Bertelsmann Foundation Health Care Monitor. Representative samples of the German population have been surveyed since 2001. Since 2011 this is conducted by the GfK, one of the largest opinion poll institutes in Germany. Eighteen to 79 year old residents were surveyed in independently drawn cross-sectional studies. The main issues were experiences in health care and attitudes towards the health care system and current health policy themes. We use the standard survey waves 18 to 21, which were conducted from 2011 to 2013. The samples were drawn randomly from an access panel (GfK Mail panel with more than 40,000 members in 2012), to obtain high responses, because its membership is composed exclusively of people, who are willing to participate in such studies [19]. A total of 1778 (response of 80.8%), 1782 (response of 81.0%), 1772 (response of 80.5%) and 1795 (response of 78.0%) people were surveyed in these waves. The participants received a written postal survey of around 30 pages [19–21].

### Variables

The types of health insurance were divided into private health insurance and SHIs. To identify differences between the latter funds, SHI respondents were also assigned to their specific SHI fund, namely AOK, BARMER GEK, BKK, DAK, TK, IKK and other SHI funds. The Health Care Monitor used a socioeconomic status measure with income, educational attainment and occupational status components, and a maximum score of 27 points. That variable was classified in 3 groups (lower class (3–10 points), middle class (11–19 points) and a upper class (20–27 points)) (as recommended by the Robert Koch-Institute) [22, 23]. The participants were questioned about their health status with 5 possible responses. We categorized them into 3 groups (poor or less good, good and very good or excellent). To describe the waiting times the participants were asked how long they had to wait in days the previous time they had made an appointment, from arrangement to consultation with their general practitioner (GP), and how long they had to wait in their physician’s waiting room. They were also asked to evaluate the waiting time for this most recent appointment with 4 possible responses (far too long, too long, acceptable and satisfied). We categorized the answers into 3 groups (far too long or too long, acceptable and satisfied).

### Statistical analysis

Respondents, who did not answer the questions on their health insurance fund and on the waiting times for their most recent consultation, were excluded from the study population. Cross-tabulations were conducted to assess the sociodemographic characteristics of the membership of the various health insurance funds, such as average age, sex distribution and socioeconomic status, of the different health insurance funds. The mean waiting time for an appointment with the GP, and in a physician’s waiting room (mean, standard deviation, median and interquartile range) and the satisfaction with these waiting times were evaluated with respect to each health insurance fund. A logistic regression model calculated the chance of excessive waiting times (dichotomous variable) with respect to health insurance fund, age, sex, region of residence, town size, health and socioeconomic status (all available and relevant variables were included without any selection method being applied). The ninetieth percentile of waiting time distribution (10 days) was chosen as cut-off between average and excessive waiting times. A sensitivity analysis was performed using the seventy-fifth percentile (4 days) as a cut-off point. Long waiting times are not necessarily associated with dissatisfaction, because some patients make an appointment in advance. Therefore, a further regression model was conducted to calculate the chance of being dissatisfied with the waiting time for appointments at the GP with respect to the health insurance fund, age, sex, region of residence, size of town, health and socioeconomic status. All analyses were performed with SAS 9.4.

## Results

### Characteristics

A total of 7127 people were surveyed, but 1509 respondents did not state their health insurance fund or waiting times for their last contact with their GP. We included 5618 respondents in this analysis of the Health Care Monitor. A total of 4805 people (85.5%) were insured by SHI funds and 813 respondents (14.5%) were insured privately. Most SHI covered participants were insured at the AOK (*n* = 1081) followed by the BKK (*n* = 1054) and the BARMER GEK (*n* = 720). The mean age was 51.6 (SD: 16.6) and varied between 47.8 (IKK) and 55.7 (DAK). The proportion of women (51.1%) varied between 30.6% (privately insured) and 58.4% (DAK) (Table 1).

**Table 1:** Characteristics of the study population

	AOK *N* = 1081	BKK *N* = 1054	BARMER GEK *N* = 720	TK *N* = 704	DAK *N* = 539	IKK *N* = 293	Others *N* = 414	Private *N* = 813	All *N* = 5618
Age (%)									
Mean (in years)	49.6	49.4	53.3	49.9	55.7	47.8	53.0	54.9	51.6
Sex (%)									
Female	53.0	54.2	55.7	53.4	58.4	56.3	53.6	30.6	51.1
Socioeconomic Status (%)									
Lower class	35.6	19.6	17.4	10.9	14.8	25.6	17.9	7.4	19.2
Middle class	57.7	64.6	64.4	60.5	70.7	62.3	69.2	48.1	61.1
Upper class	6.7	15.8	18.3	28.7	14.6	12.1	13.0	44.5	19.7
Region of residence (%)									
West Germany	72.9	82.7	75.6	81.2	81.4	52.2	75.6	90.0	78.6
East Germany (incl. Berlin)	27.1	17.3	24.4	18.8	18.6	47.8	24.4	10.0	21.5
Health status (%)									
Excellent, very good	25.3	30.5	28.3	31.1	21.9	33.6	25.1	29.2	28.1
Good	49.2	50.4	50.7	53.5	55.3	50.5	52.8	55.0	51.9
Less good, poor	25.5	19.1	21.0	15.4	22.8	15.9	22.1	15.8	20.0
Town size (%)									
Up to 10,000 residents	35.9	27.1	21.5	18.3	23.0	37.2	22.7	21.4	26.0
10,001–100,000 residents	45.2	39.4	45.3	41.8	51.4	41.0	44.2	45.6	44.1
100,001–500,000 residents	9.6	15.9	18.1	18.2	11.7	12.3	12.3	14.9	14.6
From 500,000 residents	9.3	17.6	15.1	21.7	13.9	9.6	9.6	18.1	15.3

### Waiting times

The mean waiting time for an appointment with a GP was 4.0 days (Table 2). Respondents from East Germany had to wait 6.6 days, whereas participants from West Germany waited 3.3 days. SHI insurants (4.1 days) waited only slightly longer than privately insured respondents (3.3 days). Only minor differences in waiting times were observed between the various SHIs. AOK Insurants experienced an average waiting time of 3.6 days, whereas the insurants of ‘other SHIs’ had to wait 4.9 days. Waiting times at the GP’s practice did not differ much between the various SHIs (from 29.3 min with BKK to 32.9 min with IKK). However, privately insured respondents only waited 22.4 min. Corresponding to these results, 64.5% of SHI insurants reported being satisfied with their average waiting time for appointments, whereas 77.5% of privately insured respondents were (Table 3).

**Table 2:** Average waiting times depending on health insurance

	Waiting times on appointment in days		Waiting times in physicians waiting rooms in minutes	
	Mean (SD)	Median (IQR)	Mean (SD)	Median (IQR)
Health insurance fund				
SHI (*N* = 4805)	4.1 (10.2)	2.0 (0–4)	30.8 (26.9)	25.0 (15–40)
AOK (*N* = 1081)	3.6 (8.9)	1.0 (0–3)	32.0 (27.8)	30.0 (15–40)
BKK (*N* = 1054)	4.1 (9.3)	2.0 (0–4)	29.3 (25.0)	20.0 (15–30)
BARMER GEK (*N* = 720)	4.4 (10.6)	2.0 (0–5)	30.1 (25.8)	20.0 (15–40)
TK (*N* = 704)	4.3 (9.7)	2.0 (0–5)	31.6 (27.7)	25.0 (15–40)
DAK (*N* = 539)	3.8 (7.4)	2.0 (0–4)	30.1 (24.4)	25.0 (15–40)
IKK (*N* = 293)	4.2 (9.2)	1.0 (0–4)	32.9 (29.6)	30.0 (15–45)
Others (*N* = 414)	4.9 (17.1)	2.0 (0–4)	31.5 (30.1)	25.0 (10–40)
Private (*N* = 813)	3.3 (14.6)	1.0 (0–3)	22.4 (17.7)	17.0 (10–30)
Sex				
Male (*N* = 2746)	4.4 (13.5)	1.5 (0–4)	28.8 (25.5)	20.0 (15–30)
Female (*N* = 2872)	3.6 (7.7)	1.0 (0–4)	30.4 (26.3)	20.0 (15–40)
Age groups				
18–29 (*N* = 699)	3.0 (16.6)	1.0 (0–2)	33.8 (29.9)	30.0 (15–40)
30–39 (*N* = 675)	3.1 (7.2)	1.0 (0–3)	31.9 (30.0)	15.0 (10–40)
40–49 (*N* = 1152)	3.1 (6.1)	1.0 (0–4)	28.3 (25.4)	20.0 (10–30)
50–59 (*N* = 1076)	3.4 (6.0)	1.0 (0–3)	30.0 (26.5)	20.0 (15–32.5)
60–69 (*N* = 1005)	5.1 (13.2)	2.0 (1–5)	27.8 (23.4)	20.0 (15–30)
70–79 (*N* = 1011)	5.7 (13.5)	2.0 (1–5)	28.2 (21.6)	20.0 (15–30)
Region of residence				
West Germany (*N* = 4413)	3.3 (9.0)	1.0 (0–3)	26.8 (22.9)	20.0 (10–30)
East Germany (*N* = 1205)	6.6 (16.0)	2.0 (0–7)	39.8 (32.9)	30.0 (15–60)
Town size				
Up to 10,000 (*N* = 1459)	3.1 (9.9)	1.0 (0–3)	31.0 (27.8)	25.0 (15–30)
10,001–100,000 (*N* = 2475)	4.2 (12.4)	2.0 (0–4)	29.4 (25.6)	20.0 (15–30)
100,001–500,00 (*N* = 822)	4.2 (9.5)	2.0 (0–5)	28.6 (25.2)	20.0 (15–30)
From 500,000 (*N* = 862)	4.8 (9.2)	2.0 (0–6)	28.8 (24.1)	20.0 (15–30)
Socioeconomic status				
Lower class (*N* = 1012)	4.3 (11.1)	1.0 (0–4)	31.6 (26.9)	30.0 (15–40)
Middle class (*N* = 3231)	3.8 (8.3)	1.0 (0–4)	29.2 (25.2)	20.0 (15–30)
Upper class (*N* = 1042)	4.5 (12.6)	2.0 (0–5)	27.7 (24.0)	20.0 (10–30)
Health status (%)				
Excellent, very good (*N* = 1561)	3.2 (11.6)	1.0 (0–3)	27.9 (25.9)	20.0 (10–30)
Good (*N* = 2889)	4.1 (10.5)	2.0 (0–4)	30.0 (25.3)	20.0 (15–35)
Less good, poor (*N* = 1115)	4.7 (11.3)	2.0 (0–5)	31.2 (27.2)	25.0 (15–40)
All (*N* = 5618)	4.0 (10.9)	1.0 (0–4)	29.6 (25.9)	20.0 (15–30)

**Table 3:** Assessment of waiting times depending on health insurance

	Satisfied (%)	Acceptable (%)	Too long (%)
Health insurance fund			
SHI (*N* = 4805)	64.5	27.5	8.0
AOK (*N* = 1081)	64.6	27.3	8.1
BKK (*N* = 1054)	62.6	28.8	8.7
BARMER GEK (*N* = 720)	63.6	28.4	8.1
TK (*N* = 704)	64.8	27.4	7.8
DAK (*N* = 539)	69.4	24.3	6.4
IKK (*N* = 293)	65.6	25.1	9.3
Others (*N* = 414)	63.5	29.4	7.1
Private (*N* = 813)	77.5	19.1	3.4
Sex			
Male (*N* = 2746)	67.3	26.4	6.3
Female (*N* = 2872)	65.6	26.2	8.2
Age groups			
18–29 (*N* = 699)	63.3	26.8	9.9
30–39 (*N* = 675)	64.7	24.7	10.6
40–49 (*N* = 1152)	65.4	26.4	8.2
50–59 (*N* = 1076)	66.7	26.8	6.5
60–69 (*N* = 1005)	68.1	26.3	5.6
70–79 (*N* = 1011)	69.0	26.4	4.6
Region of Residence			
West Germany (*N* = 4413)	66.9	26.6	6.5
East Germany (*N* = 1205)	64.5	25.4	10.1
Town size			
Up to 10,000 (*N* = 1459)	66.7	26.6	6.7
10,001–100,000 (*N* = 2475)	65.3	27.0	7.7
100,001–500,00 (*N* = 822)	66.8	25.4	7.8
From 500,000 (*N* = 862)	68.9	24.5	6.6
Socioeconomic status			
Lower class (*N* = 1012)	64.3	27.0	8.7
Middle class (*N* = 3231)	66.6	26.2	7.2
Upper class (*N* = 1042)	69.5	25.7	4.9
Waiting time in days			
0–1 (*N* = 2764)	82.1	15.1	2.8
2–5 (*N* = 1778)	57.9	35.7	6.4
6–10 (*N* = 614)	41.2	44.0	14.8
> 10 (*N* = 387)	33.3	35.7	31.0
Health status (%)			
Excellent, very good (*N* = 1561)	67.8	25.4	6.9
Good (*N* = 2889)	66.6	26.1	7.3
Less good, poor (*N* = 1115)	63.7	28.2	8.1
All (*N* = 5618)	66.4	26.3	7.3

### Chance of excessive waiting time

Despite the relatively homogeneous waiting times, the chance of an excessive wait of 10 days or more was unequally distributed (Table 4). A total of 10.5% of all SHI insurants and 7.5% of privately insured respondents had to wait 10 days or more for an appointment in primary care. Compared to private health insurance, the chance for an excessive waiting time ranged between an OR of 1.17 (95%-CI: 0.81–1.70) for AOK insurants and 1.64 for TK insurants (OR: 1.64; 95%-CI: 1.14–2.36). Participants aged 18 to 29 had the lowest chance of excessive waiting times. From the age of 30 upwards the chance of excessive waiting times increased with increasing age and was 183% higher (OR: 2.83; 95%-CI: 1.74–4.61) among respondents aged 70 to 79 than those aged 18 to29. Residents of East Germany had a higher chance of waiting 10 days or more than residents from West Germany (OR: 2.90; 95%-CI: 2.37–3.54). Compared to participants from towns with less than 10,000 citizens, participants from towns with more than 500,000 citizens had a higher chance of excessive waiting times (OR: 1.69; 95%-CI: 1.26–2.27). Socioeconomic and health status had no impact on waiting times.

**Table 4:** Chance for waiting times of 10 days or more

	OR	95% - CI	
Health insurance funds (Reference = Private)			
AOK	1.17	0.81	1.70
BKK	1.52^*^	1.08	2.16
BARMER GEK	1.50^*^	1.03	2.16
TK	1.64^*^	1.14	2.36
DAK	1.36	0.91	2.05
IKK	1.38	0.85	2.23
Others	1.36	0.88	2.10
Age groups (Reference = 18–29)			
30–39	1.67^*^	1.00	2.77
40–49	1.97^*^	1.23	3.18
50–59	2.06^*^	1.28	3.32
60–69	2.70^*^	1.67	4.36
70–79	2.83^*^	1.74	4.61
Sex (Reference = Male)			
Female	1.01	0.83	1.23
Town size (Reference = Up to 10,000)			
10,001–100,000	1.51^*^	1.17	1.94
100,001–500,000	1.44^*^	1.05	1.98
From 500,000	1.69^*^	1.26	2.27
Region of residence (Reference = West Germany)			
East Germany	2.90^*^	2.37	3.54
Socioeconomic status (Reference = Upper class)			
Lower class	0.79	0.58	1.08
Middle class	0.84	0.66	1.06
Health Status (Reference = Very good)			
Poor	1.07	0.80	1.43
Good	1.10	0.87	1.40

*p-value< 0.05

The sensitivity analysis showed that even the chance of waiting times of 4 days or more was unequally distributed. A total of 1508 insurants waited 4 days or more. Compared to privately insured persons, the OR for longer waiting times ranged from 1.10 (95%-CI: 0.87–1.40) for AOK insurants to 1.34 (95%-CI: 1.07–1.69) for BKK insurants. The trends for age, region of residence and town size remained the same.

A second regression model was constructed to assess the chance of being dissatisfied with waiting times (Table 5). Differences between health insurance funds were similar to the first regression model on the chance of excessive waiting times. According to health insurance, the OR of being dissatisfied ranged between 1.59 (95%-CI: 0.88–2.86) for ‘other SHI’ insurants and 2.24 (95%-CI: 1.34–4.25) for BKK insurants compared to privately insured respondents. The chance of excessive waiting times increased with age (Table 4). However, the chance of being dissatisfied with waiting times decreased with increasing age. Insurants aged 70 to 79 had a 53% (OR: 0.47; 95%-CI: 0.29–0.76) lower chance of being dissatisfied than those aged 18 to 29. Socioeconomic status had no statistically significant influence on the chance of being dissatisfied. Patients from East Germany were found to be more likely to experience longer waiting times. Corresponding to that, this patient group had a higher chance of being dissatisfied with waiting times (OR: 1.60; 95%-CI: 1.24–2.05).

**Table 5:** Chance to be unsatisfied with waiting times

	OR	95% - CI	
Health insurance funds (Reference = Private)			
AOK	1.81^*^	1.11	2.93
BKK	2.24^*^	1.40	3.58
BARMER GEK	1.99^*^	1.20	3.29
TK	1.86^*^	1.12	3.09
DAK	1.67	0.96	2.90
IKK	1.95^*^	1.07	3.55
Others	1.59	0.88	2.86
Age groups (Reference = 18–29)			
30–39	1.15	0.76	1.75
40–49	0.92	0.62	1.37
50–59	0.66	0.43	1.00
60–69	0.54^*^	0.34	0.85
70–79	0.47^*^	0.29	0.76
Sex (Reference = Male)			
Female	1.06	0.84	1.34
Town size (Reference = Up to 10,000)			
10,001–100,000	1.34^*^	1.01	1.76
100,001–500,000	1.36	0.95	1.95
From 500,000	1.08	0.75	1.55
Region of residence (Reference = West Germany)			
East Germany	1.60^*^	1.24	2.05
Socioeconomic status (Reference = Upper class)			
Lower class	1.37	0.93	2.02
Middle class	1.28	0.92	1.78
Health Status (Reference = Excellent)			
Poor	1.61^*^	1.16	2.25
Good	1.23	0.94	1.62

*p-value< 0.05

## Discussion

### Characteristics

The Bertelsmann Foundation Health Care Monitor is a representative cross-sectional survey of the population of Germany. Its characteristics are therefore comparable with official statistics. The proportion of women (51.1%) agrees with the proportion from the census of 2011 (50.9%) [24]. Most respondents were insured by the SHI funds with the most insurants (AOK, BARMER GEK, TK and DAK). However, privately insured respondents (14.5%) were slightly overrepresented.

### Waiting times

Health inequalities are large in Germany with a difference in life expectancy of about 11 years in men and 8 years in women between the richest and the poorest income groups [4]. There is some evidence that the divide between private health insurance and SHI contributes to this difference. A cohort study has found that people who switched from SHI to private health insurance were in better health than people from the same socioeconomic status group who remained in the SHI [25]. A recent review found that SHI insurants are disadvantaged, with a social gradient within the SHI for access to and utilization for the health care. No conclusive evidence for the quality of care was found [7]. However, we found that the differences between SHI and privately insured respondents in terms of waiting times for the majority of patients were rather small. These findings highlight the relatively easy accessibility of primary care with little systematic socioeconomic differences in utilisation. The strength of primary care is that it mitigates health inequalities and improves population health better than systems with an orientation towards specialist care [26]. While in Germany no gate-keeping system is in place for most patients GPs serve as first point of contact to the health care system [27]. This is especially true for people from lower socioeconomic backgrounds who might otherwise encounter barriers in accessing specialist care directly [28]. Even if no causality can yet be established, the few differences in waiting times in primary care point to its capacity to mitigate health inequalities in Germany. However, a study by Roll et al. showed decreasing waiting times with increasing household incomes in Germany [1]. Our study did not find this relationship, but did find an increasing satisfaction with waiting times with increasing socioeconomic status. This could be because we used a socioeconomic index instead the household income.

### Chance of excessive waiting time

While most respondents reported only small differences in waiting times between insurance types, the chances of experiencing a waiting time of 10 days or more revealed larger differences. Of the SHI insured respondents, 10.5% waited 10 days or more compared to 7.5% of privately insured respondents. The chance of longer waiting times increased with age. Only minor differences were found between sickness funds. Socioeconomic status surprisingly had no influence. Longer waiting times could be due to low health literacy, which occurs in around 50% of the German population [29]. Health literacy goes beyond differences in education and describes the ability to understand and assess health information, while insufficient health literacy is associated with poorer use of the health care system [30]. This might become more relevant in older age due to higher disease burden, although our analysis does not provide any details to examine the role of differences in health literacy. Barriers to access to the health care system seem to be a problem for a certain part of the population with differences in insurance status and age.

Despite the increasing chance of excessive waiting times with rising age, satisfaction with waiting times increased significantly, as well. The number of comorbidities and chronic diseases rises with greater age, which could lead to an increased need for routine appointments arranged in advance. This could explain why the chance of longer waiting times increased with age and the insurants were nevertheless satisfied with their waiting times. A further possible explanation is that satisfaction with GPs tends to increase with age [31].

Insurants living in East Germany had to wait much longer than the residents of West Germany. As described in Table 2, SHI insurants or older insurants had to wait longer than young or privately insured respondents. The average age of the residents of East Germany is higher than in West Germany. Furthermore, the proportion of privately insured citizens is lower in East Germany. However, after adjusting for age and the health insurance fund, residence in East Germany is still associated with an increased chance of experiencing excessive waiting times. Health inequalities between East and West Germany have been decreasing since the reunification in 1990. However, other adverse contextual factors including economic deprivation and a general higher disease burden affect individual health in East Germany [32]. This could be also true due to larger areas with less GPs in East Germany compared to the West, that lead to longer waiting times [33].

The chance of experiencing longer waiting times was highest in the largest cities. This is a little surprising given the greater availability of GPs within an area. However, analysis from Hamburg found greater disease burden and higher primary care utilization in poorer and more densely populated areas of the city [34]. This is not matched by the greater density of GPs in those areas and could lead to unequal waiting times at the local level.

International literature on waiting times in primary care is sparse and poorly comparable [35, 36]. Socioeconomic differences were found in Spain in the waiting times for specialist services but not in primary care [37]. Even if waiting times are introduced on purpose to provide an equitable mechanism for rationing, analysis of the English NHS has shown an income and educational gradient in waiting times [38]. However, we could not find any influence of socioeconomic status in our analysis.

### Strengths and limitations

The main strength of this study is the consistent record of health insurance affiliation. The Bertelsmann Foundation Health Care Monitor is a representative cross-sectional survey of the German resident population. However, the sample size was too small to generate robust results for the smaller health insurance funds. Our study uses a socioeconomic status index measure to examine socioeconomic differences in waiting times in primary care. However, it has been shown that the components of this index, namely income, educational attainment and occupational status, only mildly correlate with each other and are thought to have different pathways to health [39]. Differences in health-care utilization in the form of having higher chances of experiencing waiting times of 10 days or more depending on insurance status, may be more an effect of poor health literacy. Since no socioeconomic status differences were found within the insured from the same sickness fund, education differences might have been masked by the index. However, we did not assess any health outcomes in this analysis and did not know the reason for the last contact (i.e. chronic illness, acute disease or other reasons). The main differences that insurance status has on health status in older people seems mainly to be explained by income differences [40]. Unfortunately, we were only able to include 5618 respondents, because the other participants did not respond about their waiting times (*N* = 1453) or their health insurance fund (*N* = 56). However, mean age (included participants: 51.6; excluded participants: 47.5) and the proportion of females (included participants: 51.1; excluded participants: 49.3) were comparable. Furthermore the health insurance funds represented in the responsive and not responsive participants were comparable. The most common funds were the AOK (19.2% vs. 18.4%), BKK (18.8% vs. 16.1%) and BARMER GEK (128% vs. 11.5%). Because the insurants were questioned on their most recent GP visit, a recall bias might be an additional potential limitation.

## Conclusions

We were able to show that waiting times for GPs are low and homogeneous in Germany. Despite differences in socioeconomic composition no major differences between SHIs appeared, while privately insured respondents reported shorter waiting times for appointments and at their doctor’s practice. However, we did identify a group with excessive waiting times in primary care. Barriers to access to the health care system seem to be a problem for this part of the population with differences in insurance status, age, region of residence and town size. Whether duration of waiting time has any impact on health-related outcomes and how access to health care could be improved for this patient group needs further study.

## Abbreviations

GP: General practitioner
SHI: Statutory health insurance
